# Haplotyping, linkage mapping and expression analysis of barley genes regulated by terminal drought stress influencing seed quality

**DOI:** 10.1186/1471-2229-11-1

**Published:** 2011-01-04

**Authors:** Sebastian Worch, Kalladan Rajesh, Vokkaliga T Harshavardhan, Christof Pietsch, Viktor Korzun, Lissy Kuntze, Andreas Börner, Ulrich Wobus, Marion S Röder, Nese Sreenivasulu

**Affiliations:** 1 Leibniz-Institute of Plant Genetics and Crop Plant Research (IPK), Corrensstr.3, 06466 Gatersleben, Germany; 2 KWS LOCHOW GmbH, Ferdinand-von-Lochow-Str.5, 29303 Bergen, Germany; 3 Nordsaat Saatzucht GmbH, Böhnshauser Straße 1, 38895 Langenstein, Germany

## Abstract

**Background:**

The increasingly narrow genetic background characteristic of modern crop germplasm presents a challenge for the breeding of cultivars that require adaptation to the anticipated change in climate. Thus, high priority research aims at the identification of relevant allelic variation present both in the crop itself as well as in its progenitors. This study is based on the characterization of genetic variation in barley, with a view to enhancing its response to terminal drought stress.

**Results:**

The expression patterns of drought regulated genes were monitored during plant ontogeny, mapped and the location of these genes was incorporated into a comprehensive barley SNP linkage map. Haplotypes within a set of 17 starch biosynthesis/degradation genes were defined, and a particularly high level of haplotype variation was uncovered in the genes encoding sucrose synthase (types I and II) and starch synthase. The ability of a panel of 50 barley accessions to maintain grain starch content under terminal drought conditions was explored.

**Conclusion:**

The linkage/expression map is an informative resource in the context of characterizing the response of barley to drought stress. The high level of haplotype variation among starch biosynthesis/degradation genes in the progenitors of cultivated barley shows that domestication and breeding have greatly eroded their allelic diversity in current elite cultivars. Prospective association analysis based on core drought-regulated genes may simplify the process of identifying favourable alleles, and help to understand the genetic basis of the response to terminal drought.

## Background

Drought is one of the most serious abiotic stress factors which occur throughout the development of the plant and, if sufficiently severe and/or prolonged, results in the modification of the plant's physiology and severely limit crop productivity. Plants have evolved a range of defence and escape mechanisms [1], and these are typically mediated by multiple rather than by single genes. In barley, QTL underlying drought tolerance has been mapped to almost every chromosome [2-6]. However, little information has been gathered to date regarding the genomic location of drought-regulated genes, either expressed throughout plant development or at late reproductive stages influencing seed yield and quality.

Of all the genetic marker types available, single nucleotide polymorphisms (SNPs) are the most abundant, and thus offer the greatest level of genetic resolution. They are of potential functional relevance and they are also well suited to high throughput analytical methods [7]. The representation of SNPs on the barley linkage map has grown over recent years [8-10], and in particular, a SNP-based map featuring gene sequences expressed differentially in response to various abiotic stresses has recently been developed [7]. Here we present a SNP-based genetic map of barley, specifically focussing on nucleotide variation in ESTs demonstrated to be involved in the response of barley to drought stress occurring at early vegetative stages, during anthesis and the grain filling process.

While the productivity of the cereals has risen greatly since their domestication, in response to farmer selection and methodical breeding, there are indications that the increasing fixation of elite alleles in modern breeding germplasm is already inhibiting further genetic gain. In the face of potential climate change, these elite allele combinations may become sub-optimal and will necessitate a search for better adapted alleles among crop landraces or wild materials [11]. Population of wild barley (*Hordeum vulgare *ssp. *Spontaneum*, hereafter referred to as *H. spontaneum*) have been shown to possess favourable genetic variation for a number of agronomic traits [12,13] including biotic [14,15] and abiotic stress tolerance [2,16-19].

We report haplotyping data for 17 starch biosynthesis/degradation genes demonstrating the broad diversity among *H. spontaneum *accessions and *H. vulgare *landraces but rather limited genetic variance in the current elite breeding germplasm by fixing certain haplotypes. Similar observations were made for seed starch accumulation during terminal drought for a diverse set of 50 barley accessions.

## Results and Discussion

### SNP discovery in sequences responding to drought stress

The initial set of 613 drought-responsive ESTs (covering 20 functional categories; Additional file 1) was determined from 5 or 21 day old seedlings, flag leaves-post anthesis or developing grains. Suitable sequence information from the four parents of mapping population and the four advanced backcross (AB) population parents were obtained for 327 genes (53.3%). The sequence reads were assembled individually for each locus. A total of 1,346 informative SNPs were dispersed through 263 of the sequences, giving a mean SNP density of 5.1 per kb (Additional file 2). The Oregon Wolfe parents were the best differentiated (627 SNPs across 181 ESTs, density 3.4 per kb), which is consistent with comparisons made elsewhere between these two lines [7,20]. Some 30% of the loci were polymorphic between cvs. Steptoe and Morex, as noted in the previous studies for these cultivars [10,20]. The proportion of informative loci in cv. Brenda versus HS584 was 33%, and between cv. Scarlett and ISR42_8 39%. Note that a polymorphism survey based on 400 microsatellite loci showed that 46% were informative between cv. Brenda and HS584 [21], while 97 out of 220 (44%) were polymorphic between cv. Scarlett and ISR42_8 [22].

### Marker development and linkage mapping

**Figure 1:**
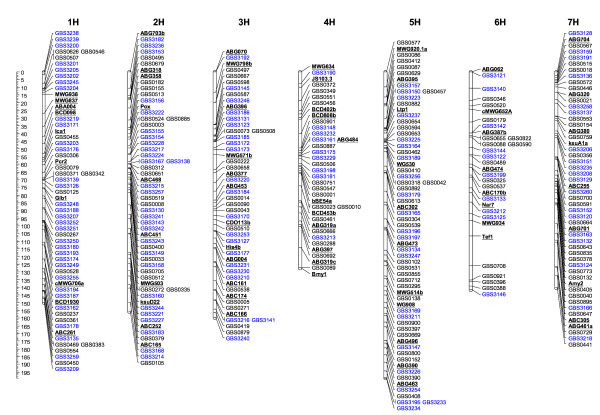
**A combined barley genetic map of EST-based SNPs segregating in the Steptoe/Morex and/or the Oregon Wolfe mapping population**. *De novo *markers (blue) were integrated with previously mapped SNP loci (black) and common BIN markers (black and underlined).

The SNPs present in the 263 ESTs were converted into 31 pyrosequencing-based markers for Steptoe/Morex, 76 for Oregon Wolfe and 34 markers common to both populations, for a total of 141 SNP markers (Table 1). Of the 20 functional gene categories represented among the 613 initially selected ESTs, 17 classes were retained among the genes tagged by the 141 markers (Additional file 1). Genes involved in carbohydrate, amino acid metabolism, hormone signalling, storage protein synthesis and the response to desiccation, as well as a number of transcription factors were particularly common (Additional file 1). Genotypic data associated with both the 141 *de novo *SNP markers (GBS3120-GBS3260), and with an established set of 140 GBS (GBS0001-GBS0921; [10]) and 71 BIN markers were then used to construct a 352 marker-based map (Figure 1), in which the BIN markers were situated as expected [10,23]. The only change in GBS marker order occurred on chromosome arm 3HL, where GBS0538 mapped distal, rather than proximal to ABC161 [10]. The genetic length of each chromosome ranged from 127.2 cM (4H) to 198.8 cM (5H), and the overall map length was 1,072 cM (Table 1). Given the unequal genomic distribution of the marker loci, marker development was focussed on chromosomes 1 H (32 loci) and 2 H (28 loci), because these chromosomes are known to harbour drought-related QTL (unpublished data and [3,4,6]). For example, Teulat *et al*. [4] identified a QTL for drought related traits at the SSR marker Ebmac684 on 2 H analysing grain material from field grown barley from an environment with limited water availability especially during the grain filling period. The marker Ebmac684 maps close to ABC468 [24], in a chromosomal region where several *de novo *markers representing putative candidate genes were mapped. These genes encode transcription regulators (GBS3215, GBS3217, GBS3224), a cytochrome protein (GBS3138), a protein kinase (GBS3167) and the starch branching enzyme (GBS3257). Chromosomes 4 H (nine loci) and 6 H (ten loci) contained the least *de novo *marker, while 21, 22 and 19 loci were mapped to chromosomes 3 H, 5 H and 7 H, respectively. Each member of the pairs of sequences GBS3141/GBS3216, GBS3193/GBS3250, GBS3129/GBS3260, and GBS3150/GBS3223 was derived from the same EST, and thus mapped to the same position (Additional files 3 and 4). The pairs GBS3230/GBS3231, GBS3172/GBS3173 and GBS3154/GBS3155/GBS3228 each are based upon different EST clusters but represent the same gene as they do not overlap due to shorter contigs, and mapped to a single chromosome bin (Additional files 3 and 4).

**Table 1:** Marker frequency and map length of the individual mapping populations for deriving the integrated map.

	SM		OWB		integrated	
**Chromosome**	**Marker**	**cM**	**Marker**	**cM**	**Marker**	**cM**

1H	13	148.7	24	148.8	32	149.7

2H	15	135.8	22	155	28	155.3

3H	12	135.2	14	178.7	21	159.2

4H	3	107.4	8	123.2	9	127.2

5H	10	154.6	18	202.1	22	198.8

6H	5	96.9	8	104.6	10	141.4

7H	7	135.4	16	154.1	19	140.1

total	65	914	110	1066.5	141	1071.7

### Overlap with other barley SNP maps

Only seven of the previously mapped abiotic stress related barley genes belong to the present map of drought-responsive 141 *de novo *SNP markers [7] (Additional file 4). GBS3193 and GBS3250 belong to the same mapped abiotic stress marker scsnp04853, mapped to chromosome 1 H in [7]. On chromosome 2 H, GBS3244 is covered by scsnp00592, GBS3138 by scsnp01644 and GBS3158 by scsnp03343. GBS3198 (chromosome 4H) corresponds to scsnp06435, and GBS3247 (chromosome 5H) to scsnp14350. Six of the seven overlapping markers mapped to their expected chromosomal BIN, but GBS3244 appeared to lie proximal, rather than distal to ABC252. Taken the consensus transcript map in [10] five of the *de novo *SNP loci are represented there, namely GBS3178/GBS0237 (chromosome 1H), GBS3158/GBS0400 (chromosome 2H), GBS3246/GBS0073 and GBS3170/GBS0043 (chromosome 3H), and GBS3128/GBS0018 (chromosome 7H). A further 14 GBR or GBM markers identified the same loci as the *de novo *SNPs, but two (GBS3139 on chromosome 1 H, GBR1494 on chromosome 2H; and GBS3207 on chromosome 1 H, GBR1571 on chromosome 2H) had a discrepant chromosome location. The pairs GBS3253/GBR0625 and GBS3185/GBM1405 all mapped to chromosome 3 H but to different bins (Additional file 4). Another high-density transcript linkage map based on a total of 2890 SNP, CAPS and INDEL markers was published by Sato *et al*. [9]. According to unigene IDs, 31 GBS markers show overlap with 28 loci of the present map. Finally, 67 of the 2,943 SNP loci present on the Close *et al*. [8] map correspond to GBS marker(s), with no discrepancies in terms of chromosomal location. Marker 1_0686 (matching GBS3207 and GBR1571 [10]) was located to chromosome 1 H, thereby confirming the position of GBS3207. In summary, 52 of the 141 *de novo *SNP loci of drought-responsive genes represent novel means for characterizing the genetic basis of drought tolerance in barley and they may also provide useful information for the construction of the barley physical map as the next step towards genome sequencing.

### The drought stress response of mapped transcripts over development

**Figure 2:**
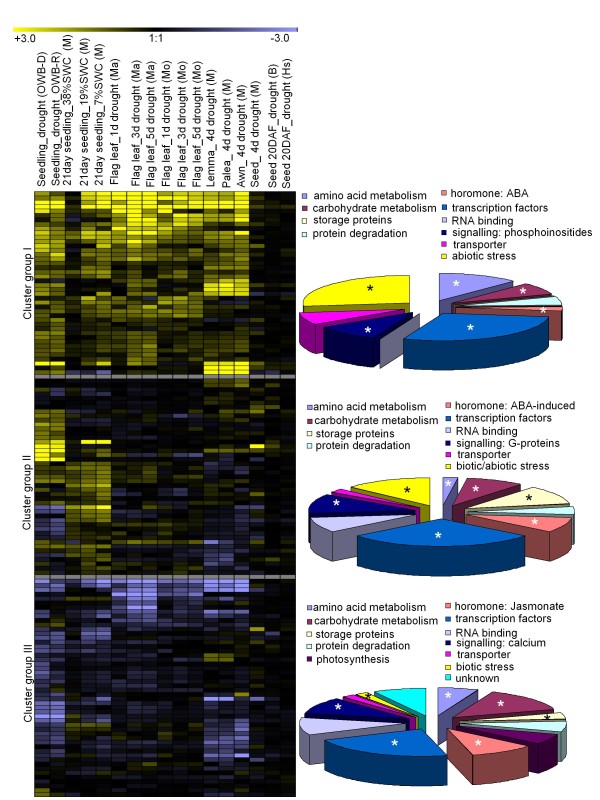
**Expression profiles of barley genes responsive to drought**. Expression ratios (drought vs control) are colour-coded: dark yellow >6 fold up-regulated, black no change, violet >6 fold down-regulated. The proportion of genes within a given functional transcript group is shown in the corresponding pie chart on the right with significantly overrepresented gene categories marked by star symbol. Each gene is represented as horizontal row (for order, see Additional file 3) and developmental stages are detailed in the vertical columns (d: days of exposure to drought and %SWC: soil water content). Gene expression data refer to cvs. Brenda (B), Morex (M), Morocco (Mo), Martin (Ma), Oregon Wolf Barley-Dominant (OWB-D), Oregon Wolf Barley-Recessive (OWB-R), Hs (*H. spontaneum *HS584). Expression data from individual replications are given in Additional file 3.

To reveal the drought stress response of mapped transcripts during various stages of development, we normalized the expression data by utilizing the publicly available expression data sets deposited in Gene Expression Omnibus (GEO) from five (GEO accession series number: GGSE3170) and 21 (GSE6990) day old seedlings, flag leaves post anthesis (GSE15970), green spike tissues (awn, lemma and palea, GSE17669) and own data from developing grain during 20 days after fertilization (DAF). A range of barley cultivars has been used to generate these data, including the drought tolerant cv. Martin and the susceptible cv. Moroc9-75, parents of mapping and AB populations (OWB-D, OWB-R, Morex, Brenda and Hs584). The clustering process identified three major groups: groups 1 and 2 contained genes which are up-regulated as a result of drought stress, while the ones in group 3 were down-regulated (Figure 2). While group 2 genes showed up-regulation mostly in early vegetative tissues, group 1 members were up-regulated across all developmental stages, and were expressed in a range of organs (seedlings, flag leaf, lemma, palea, and awn and to a lesser extent in the developing grain). Thus, group 1 genes could be considered to represent a core set of drought responsive genes. The functional groups particularly overrepresented in groups 1 and 2 included transcription regulators, genes induced by abiotic stress, genes responsible for the synthesis of storage proteins and genes related to amino acid and carbohydrate metabolism, and ABA-induced hormone related genes, calculated by Fisher's exact test with a P-value cut off 0.01 (Figure 2 and Additional file 3).

#### Regulators

**Figure 3:**
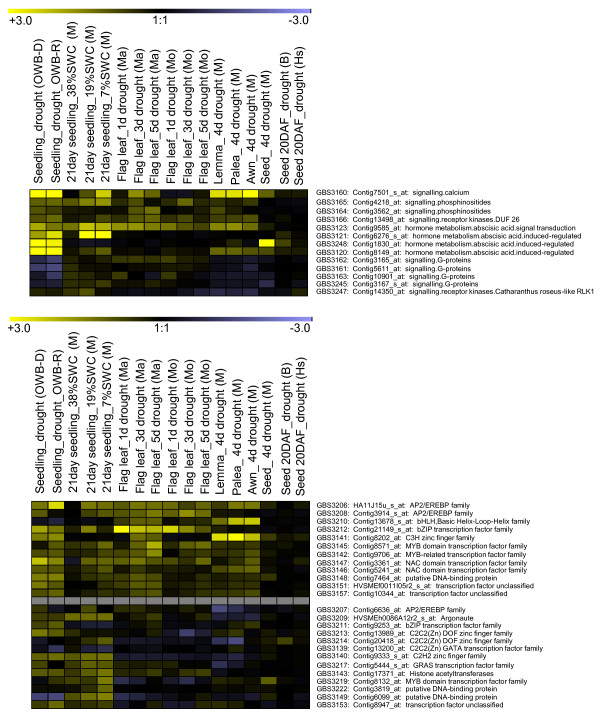
**Expression profiles of mapped barley genes up-regulated by drought stress**. Upper panel: hormone and signalling genes, lower panel: transcription factor families. For abbreviations, see Figure 2 legend. Expression data from individual replications are given in Additional file 3.

An ABA signalling gene (protein phosphatase 2C, marker GBS3123), a bZIP ABA-responsive element binding protein (GBS3212) were consistently up-regulated by drought throughout development in barley (Figure 3). In *A. thaliana*, protein phosphatase 2C regulates a Snf1-related kinase [25], and mediates signal transduction to an ABF2 transcription factor [26]. Thus in barley, it seems likely that an ABA signalling pathway orchestrates the adaptive response to drought, not just at the seedling stage but also in the flag leaf, awn, lemma and palea (Figure 3). In addition several Ras family G-proteins (GBS3161, GBS3162, GBS3163, GBS3245) thought to be involved in ABA signalling are found to be induced in 21 day seedlings and flag leaf (Figure 3). Several ABA-induced late embryogenesis abundant proteins (GBS3120, GBS3121, GBS3248) were induced to drought in seedlings (Figure 3), and these have been shown previously to be involved in desiccation tolerance [27]. A number of ABA signalling related genes were included in the genetic map (Additional file 3). Other transcription factors were induced by drought in a non-organ specific manner; these included AP2/ERF II (GBS3206), VII (GBS3208), VIII (GBS3207), bHLH (GBS3210), bZIP (GBS3212, GBS3211), MYB (GBS3142, GBS3145, GBS3219), NAC (GBS3146, GBS3147) and several other unclassified factors (Figure 3). The specific function(s) of most of these regulators remains unclear, but their up-regulation by drought stress indicates that they probably do play a role in the plant's response to water deficit.

#### Abiotic stress induced genes

Genes encoding dehydrin 9, universal stress proteins, hydrophobic proteins and various classes of heat shock proteins (HSPs) were induced by drought across all the developmental stages (Figure 2 group 1). Among the HSPs were HSP70 (GBS3180); HSP81-1 (GBS3182) and HSP26 (GBS3181), which mapped, respectively, to chromosomes 1 H, 2 H and 4 H (Additional file 3). Other HSPs were not so generally up-regulated by drought. The up-regulation of HSP is consistent with their presumed protection of proteins from oxidative damage induced by drought stress [28].

### Drought response of mapped transcripts contributing to seed quality

**Figure 4:**
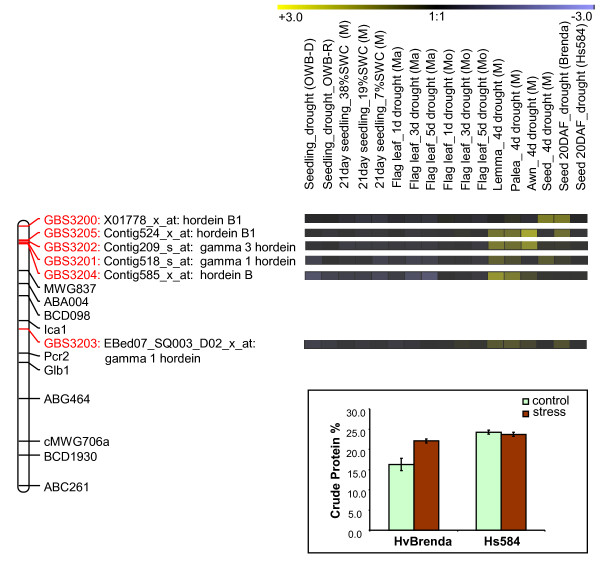
**The cluster of sulphur-rich hordein genes on the short-arm barley chromosome 1 H (left panel) and their corresponding expression profiles during development**. For abbreviations, see Figure 2 legend. Expression data from individual replications are given in Additional file 3. In the lower panel, percent crude protein estimated based on seed nitrogen (N%) for the two parents of introgression line population (*H.vulgare *Brenda and *H. spontaneum *584) from control and drought stress treatments is presented.

Barley grain storage proteins comprise a mixture of four distinct prolamin polypeptides: the B- and γ- (sulphur-rich) hordeins, the C- (sulphur-poor) hordeins and the high molecular weight D-hordeins. The hordein genes are known to be organised in clusters encoding the B-hordeins (*Hor2 *and *Hor4*), C-hordeins (*Hor1*), γ-hordein (*Hor5*) and D-hordein (*Hor3*) which are all located on chromosome 1 H [29]. The present genetic map showed that GBS3200, a marker for B1-hordein, lay near the telomere of chromosome 1 H, while GBS3205 (marking another B1-hordein) was linked closer to GBS3202 (B3-hordein), around 11 cM distant from GBS3201 (γ1-hordein). A third B-hordein marker (GBS3204) was placed further apart, closer to γ3-hordein. Thus the B-hordein gene family is represented by at least three different loci on the short arm of chromosome 1 H, while the γ-hordein genes also map to two distinct loci on the same chromosome arm (Figure 4).

The regulation of hordein family gene transcription includes DNA methylation [30,31] and the concerted action of distinct transcription factor families [32,33]. The expression of all the sulphur-rich hordein genes was promoted by drought in the awn, lemma and palea (Figure 4). Hordein transcripts first appear in the endosperm at 12 days post anthesis, peaking in expression by 16 days, and then maintaining this level until grain maturity [34,35]. The B1-hordein genes were induced in developing seeds by drought stress in cv. Brenda, but less prominently so in HS584 (Figure 4), indicating distinct differences in B-hordein gene expression between cultivated barley and its wild relative. Correspondingly, the seed nitrogen/protein content also increased under drought in Brenda but not in HS584. However, the absolute levels remained high in the control plants (Figure 4).

**Figure 5:**
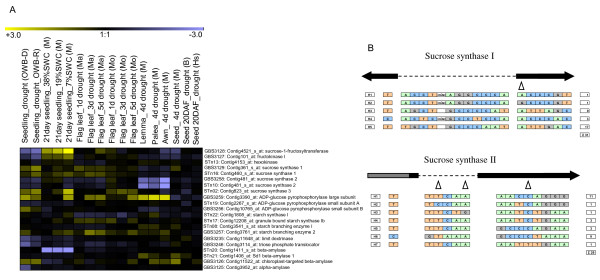
**The expression profiles of a selection of starch biosynthesis/degradation genes responsive to drought during development (panel A)**. For abbreviations, see Figure 2 legend and expression data from individual replications are given in Additional file 3. The location of SNPs and the resulting haplotypes (H) present in both sucrose synthase types I (GBS3129) and II (GBS3258) genes are given in panel B. Black arrows indicate exonic regions and grey bars untranslated regions. Introns are represented by dashed lines. Shown below are the haplotype groups with the respective polymorphisms and the number of lines per group. Triangles indicate accession-specific SNPs. Haplotypes of all the genes detailed in Additional file 5. Correlation of seed starch content under drought to specific haplotypes of sucrose synthase type II is given in Additional file 6.

In contrast, the down-regulation of the gene family members of key starch biosynthesis genes, sucrose synthase, ADP-glucose pyrophosphorylase are down-regulated by terminal drought stress in the post anthesis period during 20 DAF (Figure 5A). Several genes associated with the activity of the starch branching enzyme became activated by terminal drought stress, which has implications for the synthesis of amylopectin. Certain genes involved in starch degradation (e.g., those encoding sd1-ß-amylase and chloroplast-targeted ß-amylase) were also induced by drought stress, which points to a concerted fine tuning of starch biosynthesis and degradation in impairing seed starch accumulation and seed quality. However, many genes associated with carbohydrate metabolism including the genes encoding sucrose synthase type I (GBS3129), ADP-glucose pyrophosphorylase large subunit (GBS3259) and starch branching enzyme class II (GBS3257) were up-regulated by drought stress in seedlings, the flag leaf, the awn, lemma and palea (Figure 5A). The production of starch in vegetative tissues of *Arabidopsis thaliana *has been found to be negatively correlated with plant biomass [36]. Likewise, we might expect that starch accumulation in vegetative tissues negatively affects plant growth under drought stress.

### Haplotype analysis of carbohydrate metabolism genes

A detailed analysis of sequence variants within 17 starch biosynthesis/degradation genes was conducted for a core set of 32 accessions, which included landraces, elite breeding lines, the mapping population parents and *H. spontaneum*. This delivered 180 polymorphic sites (SNPs and indels) across both intronic and exonic sequence, and led to the recognition of 78 haplotypes (Table 2). Overall the elite breeding lines, including cv. Brenda, showed little haplotypic variation, but the remaining materials fell into a number of haplotype groups indicating broader genetic diversity. Figure 5B summarizes the variation present within the genes encoding sucrose synthase types I (CR-EST:HY09D18, marker: GBS3129) and II (CR-EST:HA31O14, CR-EST:HF08A21; GBS3258) whereas the haplotyping data for the remaining genes are listed in Additional file 5.

**Table 2:** Haplotype details for the core set of starch biosynthesis/degradation genes.

EST	BLAST search result	Number of haplotypes	SNPs	InDels	Approx. sequence length (bp)
HY09D18	Sucrose synthase 1	5	18	1	360

HF08A21	Sucrose synthase 2	7	14		550

HA31O14	Sucrose synthase 2	5	4		750

HF21A17	Hexokinase	5	4		350

HY04O18	ADP-glucose pyrophosphorylase large subunit	5	4		350

HB16O10	ADP-glucose pyrophosphorylase small subunit (alternatively spliced)	4	5		1050

HA31F12	ADP-glucose pyrophosphorylase small subunit	3	2		1000

HB05N09	Starch synthase I	2	1	1	900

HF05C15	Starch synthase IV	4	9		650

HY09J12	Granule-bound starch synthase 1b	4	5		350

HB30O07	Starch branching enzyme I	10	45	8	550

HB21K16	Starch branching enzyme IIa	2	1		550

HZ53C02	Beta-amylase	3	2		450

HF17A10	Beta amylase	4	3	1	280

HF11O03	Sd 1 beta-amylase	5	5		280

HZ60P11	Alpha glucosidase	4	35	3	1800

HB20O07	Gamma 2 hordein	6	9		300

	Σ	78	166	14	

Within the 360 bp re-sequenced region of the sucrose synthase type I amplicon, 18 SNPs and a 3 bp indels were found. Among the SNPs, 11 were situated within an intron and seven (six synonymous) within an exon; the single non-synonymous SNP was a transition variant present in cv. Morex, which converted a glycine residue to a serine. The accessions could be classified into five haplotypic groups (H1-H5), the largest of which (H5) included all the elite breeding lines and half of the remaining *H. vulgare *accessions. H2 contained only one entry (cv. Morex), as did H1 (HS584). H3 captured several *H. vulgare *and the other *H. spontaneum *accessions, as well as the Oregon Wolfe dominant parent. The Oregon Wolfe recessive parent fell into H4 along with two other *H. vulgare *lines (Additional file 5).

GBS3258 represented about 550 bp of the sucrose synthase type II sequence, and the re-sequencing of 29 accessions generated 14 SNPs. These allowed the recognition of seven haplotypes (H1-H7), of which H2, H3 and H7 each contained only one accession. The elite breeding lines were split among the two major groups H1 and H6, along with most of the *H. vulgare *accessions, although H6 also included ISR42-8, an *H. spontaneum *accession. Groups H4 and H5 each contained three accessions, the former containing the remaining *H. spontaneum *accessions, and the latter the remaining *H. vulgare *ones.

The relatively high level of haplotype diversity in these two sucrose synthase genes among non-elite lines suggests that these genes have experienced selection processes during the course of domestication and farmer's selection. However, for improving sink strength specific haplotypes (H5 from sucrose synthase I, H1 and H6 from sucrose synthase II) were fixed in the elite lines during the breeding. In maize, key starch biosynthesis enzymes and soluble carbohydrates were measured from field grown samples from hundred recombinant inbred lines and revealed major QTLs close to the locus sucrose synthase (*Sh1*) gene known to be linked to improved starch accumulation [37]. To confirm the importance of *Sh1 *locus, sucrose synthase gene polymorphisms was analyzed in 45 genetically unrelated maize lines. Therein, the *Sh1 *locus was also found to significantly associate with higher starch and amylase content as well as grain matter from multi-location field trials [37]. In the present study also a high level of allelic diversity was detected in the genes encoding sucrose synthase I, sucrose synthase II, starch branching enzyme I and α-glucosidase, while the genes encoding both the small and large subunits of ADP-glucose pyrophosphorylase were rather non-polymorphic (Additional file 5).

Haplotype variation was also used to estimate the extent of the genetic separation between cv. Brenda and HS584. Among the 13 informative sequences, three harboured non-synonymous exonic SNPs. Two neighbouring SNPs within the granule bound starch synthase Ib gene [CR-EST:HY09J12] were present in both HS584 and a number of the barley accessions, while the SNPs present in both the ß-amylase [CR-EST:HF11O03] and the γ-2 hordein [CR-EST:HB20O07] genes were unique to HS584. Another four genes (sucrose synthase type I [CR-EST:HY09D18] and type II [CR-EST:HA31O14, CR-EST:HF08A21], ADP-glucose pyrophosphorylase small subunit sequence [CR-EST:HB16O10], and starch branching enzyme I [CR-EST: HB30O07]) were found to contain synonymous exonic substitutions. Intronic SNPs were also detected in most of the genes, including the ADP-glucose pyrophosphorylase small subunit sequence [CR-EST:HB16O10], a gene known to undergo alternative splicing [38]. These data confirm that wild barley alleles own the capability to alter protein sequences (non-synonymous SNPs), codon usage (synonymous SNPs) and the splicing process (intronic SNPs) and emphasize the potential of the Brenda/HS584 introgression line population to serve as a model for the investigation of favourable wild barley alleles.

### Intraspecific variation of grain starch content under terminal drought

**Figure 6:**
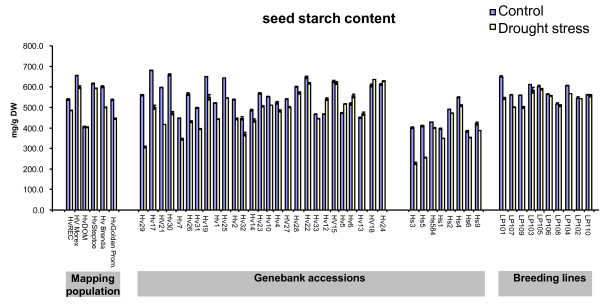
**Variation for seed starch content in 50 barley accessions**. Seed starch content measured from mature grain of control and drought stress, which is expressed in mg/g dry weight (DW). Hv: *H. vulgare*, Hs: *H. spontaneum*. The breeding lines encoded "LP" represents yet unreleased varieties bred by Lochow-KWS. Further accession details are provided in Additional file 8.

**Figure 7:**
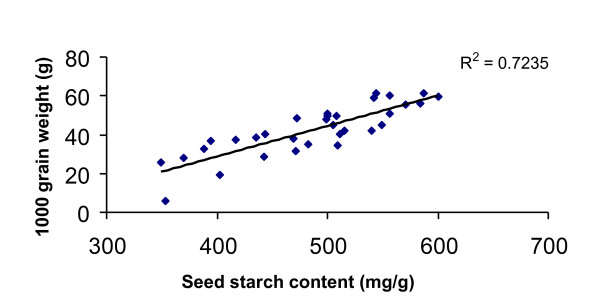
**Scatter plot and correlation analysis of seed starch content and thousand grain weight (TGW) under terminal drought stress**. For further details refer 'Methods' section.

Identifying the molecular basis of phenotypic variation can provide improved insights into the mechanisms responsible for key agronomic traits such as grain yield stability. Thus patterns of starch accumulation during terminal drought were monitored for a diverse set of 50 barley accessions. A high genetic variation for grain starch content was observed (Figure 6). The starch content of the non-stressed barley landraces varied from 450-680 mg/g dry weight, while among the elite breeding lines, the range was 514-648 mg/g (Additional file 5 and Figure 6). Within gene bank accessions of *H. vulgare *and *H. spontaneum*, two major classes were found; one class suffered a reduction of up to 45% in the amount of starch accumulated under terminal drought conditions, whereas the other performed well in both well-watered and terminal drought conditions (Figure 6). Unlike the wild barleys and the landraces, the sample of elite breeding lines showed little variation for starch accumulation, although many performed well under terminal drought stress. Three accessions (LP101, LP107 and LP109) suffered a slight reduction in grain starch content and, consequently, thousand grain weight (TGW) when challenged with terminal drought stress under both field and green house conditions (Additional file 5). Interestingly, those lines which showed dramatic reduction of starch content under terminal drought in comparison to their respective controls possess haplotypes H3 (Hv32), H4 (Hs3, Hs5, Hv10) and H5 (OWB-DOM, Hv29, Hv30) from sucrose synthase II gene (starch content of control versus stress with low correlation of R^2 ^= 0.4) and lines possessing haplotype H6 (ISR42_8, Hv13, Hv20, Hv22, LP103, LP104, LP106, LP107, LP110) from sucrose synthase II gene correlate positively to optimum starch accumulation under both control and drought treatments (with R^2 ^= 0.9 at a significance level of α = 0.01 using Steiger's Z-test for Pearson correlation) [Additional file 6]. Similarly, we also noticed a higher genetic variation for TGW of barley landraces not only under control conditions but also under drought stress (Additional file 7). Moreover, global correlation analysis between seed starch content and an average of TGW obtained from multi-location field trials from two consecutive years (2007 and 2008) using both methods (water withhold and potassium treatments) and green house screening for all genotypes under drought stress conditions signifies correlation with R^2 ^= 0.72 at a significance level of α = 0.01 using Steiger's Z-test for Pearson correlation (Figure 7). The origin and IG-number is provided for all 50 barley accessions in Additional file 8.

## Conclusions

The genetic mapping of 141 drought regulated ESTs has extended the abiotic stress SNP map of barley [7] by a further 134 novel markers. An extensive expression analysis of these ESTs at various developmental stages for drought response and across a range of barley accessions resulted in creating an expression map for genetically mapped markers. The mapped candidate genes have been reported to co-segregate with drought related traits, which fall into diverse functional categories like stress response (e.g. dehydrin [39,40]), transcription factors (e.g. CBF [5]), carbohydrate metabolism (e.g. sucrose synthase [3]) and many more [3,6,41,42]. The map also disclosed an interesting correlation between several clusters of sulphur-rich hordeins on the short arm of chromosome 1 H and their co-expression, potentially linked to methylation based regulation [30,31].

The haplotype structure of 17 starch biosynthesis/degradation genes was explored, revealing that the genes encoding sucrose synthase (both types I and II) and starch synthase were surprisingly variable in wild barley and landraces. Superior alleles related to haplotype H5 from sucrose synthase I and H6 from sucrose synthase II were found to be present in the studied breeding lines too, selected for improved performance. This observation provides additional evidence that these alleles may be causally associated with improved starch accumulation under control as well as terminal drought stress conditions. The gained knowledge represents a valuable source for the development of functional markers to assess larger collections of barley accessions for the correlation of relevant haplotypes of starch biosynthesis/degradation genes to seed starch content under drought and, therefore, for further improvement of barley cultivars in terms of improved grain weight.

## Methods

### Plant material, starch and DNA extraction

The eight barley accessions from which ESTs were re-sequenced were the parents of mapping populations cvs. Steptoe and Morex [43], the parents of the Oregon Wolfe population [44] and the parents of AB populations cv. Scarlett and ISR42_8 [22], and cv. Brenda and the *H. spontaneum *accession 584 [21]. The Steptoe/Morex and Oregon Wolfe mapping populations comprised 80 and 94 individuals, respectively. Total genomic DNA was extracted from 4-6 g young leaf material, using the protocol described in [45].

A set of 50 barley accessions was assembled from the IPK Gatersleben and the ICARDA genebanks, and these, along with cv. Brenda and *H. spontaneum *accession 584 (HS584), were grown till flowering under a 16 h light/20°C, 8 h dark/15°C regime. Terminal drought stress was imposed for a period of three weeks beginning one week after fertilization (8 DAF) during the post-anthesis period. The automatic watering procedure was monitored by a DL2e data logger (Delta T) with SM200 sensors connected to individual pots. This enabled to maintain the control plants at 60% soil moisture and drought stressed plants at 10% soil moisture. Mature seeds were harvested from the mature plants of control and drought stressed plants and estimated TGW using MARVIN seed counter. For each line, three independent replicates were maintained for both control and drought stress treatments and for each replicate seeds were pooled from five plants.

Starch was extracted from ground mature grains in 80% v/v ethanol at 60°C, each followed by a centrifugation (15 min, 14,000 g). The final supernatant was discarded and the remaining pellet used for the quantification of starch content [46]. Starch content is measured from three replicates.

Crude protein content (%) was obtained by multiplying seed N% with the factor 5.83 [47]. Total seed N% was measured using elemental analyzer (Vario EL; Elementar analyse system).

All 50 accessions were also subjected to drought stress in the field at breeding station, Nordsaat Böhnshausen over the two consecutive years (2007 and 2008) by following two different strategies. Strategy I: All genotypes were planted as two-row plots per entry with two replications in randomized blocks, directly on soil in a closed green house/rain shelter which completely protects rain fall. Control plants were irrigated four times during the period from seed set until seed filling. For imposing terminal drought stress, ten days post anthesis staged plants were subjected to water withhold by stopping irrigation until the end of grain development. Strategy II: All genotypes were planted as three-row plots per entry with two replications in randomized blocks. Control plants remained untreated. For mimicking drought stress treatments 10% w/v potassium iodide is sprayed to whole plant at ten days post anthesis. After reaching maturity, all the genotypes of the two replicates from two strategies were harvested by hand and TGW and seed quality was determined in Nordsaat seed quality laboratory.

Correlation analysis was carried out between TGW data and starch content under drought stress. To consider seasonal variability, lines in each year were z-score normalized, and then variance was calculated across the years. The Z-score normalized TGW data for all accessions are shown as heat maps (Additional file 7). Those lines with too high variance levels were excluded from the correlation analysis. For the remaining lines, average values of TGW data were calculated across the years (2007 and 2008) from field and rain shelter as well as green house experiments. These averaged TGW data were correlated with the corresponding average values of green house replicates of seed starch content from drought treatments using the Pearson correlation measure. Statistical significance of the calculated r^2 ^values was assessed using Steiger's z-test at a significance level of α = 0.01 [48].

### SNP discovery and detection

For the sequences identified in the CR-EST database (clustering project g03) http://pgrc.ipk-gatersleben.de/cr-est/, GeneRunner software http://www.generunner.net was applied to design PCR primer pairs each amplifying a 300-600 bp fragment from an individual EST. Each 50 μl PCR contained 50-100 ng genomic DNA template, 1.5 mM MgCl_2_, 0.2 mM dNTP, 10 μM of each primer and 1U Taq DNA polymerase. After an initial denaturation of 96°C/2 min, the reactions were cycled 14 times through 94°C/30 s, 72°C (-1°C/cycle)/20 s and 72°C/90 s, and then a further 27 times through 94°C/30 s, 58°C/20 s and 72°C/90 s, before a final extension step of 72°C/3 min. After checking for correct amplification, each reaction was then purified using a MinElute™96 UF PCR Purification kit (Qiagen, Hilden, Germany) according to the manufacturer's instructions, and subjected to cycle sequencing from both ends using the relevant PCR primers. Cycle sequencing was performed with the BigDye Terminator v3.1 ready reaction cycle sequencing kit on an ABI 3730 × 1 sequencer (Applied Biosystems). The re-sequenced ESTs were aligned using the SeqMan tool within the Lasergene software package http://www.dnastar.com to identify SNPs. According to the annotation of polymorphisms, haplotype groups were determined for a core set of starch biosynthesis/degradation genes (Table 2).

SNP detection was carried out by pyrosequencing. Corresponding assays were designed with the Pyrosequencing™Assay Design Software Version 1.0.6 (Biotage AB, Uppsala, Sweden). Genomic DNA was amplified as above, except that one primer was biotinylated, and the extension step was shortened from 90 s to 30 s. Streptavidin Sepharose™High Performance (GE Healthcare Biosciences, Uppsala, Sweden) was used to obtain single stranded amplicons. SNP genotyping was performed using the PSQ HS 96A System (Biotage AB, Uppsala, Sweden). Further information on template preparation and the pyrosequencing protocol can be found in [49].

### Linkage mapping

JoinMap^® ^v4 [50] was used to construct a genetic map based on a combination of the *de novo *Steptoe/Morex and Oregon Wolfe population SNP and already published genotypic data [10]. Recombination fractions were converted to cM using the Kosambi mapping function, with the following JoinMap settings: minimum LOD score = 1.0, recombination threshold = 0.4, ripple value = 0 1 and jump threshold = 0 5. For chromosomes 3 H and 6 H, the marker order of the reference map [10] was chosen as the starting order.

### Affymetrix BarleyI GeneChip analysis

To identify drought regulated gene sets at various stages of development, Affymetrix chip CEL files derived from both control and drought-treated seedlings (Series GSE3170), 21 day old plants (Series GSE6990), flag leaves (Series GSE15970) [17], awn, lemma, palea, and the early stages of the developing grain (Series GSE17669) [51] were downloaded and merged with in house expression data obtained from developing grain 20 DAF from cv. Brenda and HS584. RNA was obtained from two independent replicates. For each replicate seeds were pooled from five plants. The developing grain harvested from the central part of the spike from both control and drought treated plants of cv. Brenda and HS584 according to [52]. The RNA was isolated using the TRIzol reagent (Invitrogen GmbH, Karlsruhe, Germany) and RNAeasy columns (Qiagen, Hilden, Germany). Probe synthesis, labelling and hybridization were performed according to the manufacturer's protocols (Affymetrix). The expression of 22,000 genes extracted from all experiments was subjected to RMA normalization, applying a linear model via the limma package using R/Bioconductor functions in Robin software [53]. After normalization, log2 expression values were derived to generate fold differences between non-stressed and drought stressed organs from independent experiments. A nested multiple testing strategy was applied, using the Benjamini-Hochberg P-value correction (P-value cut-off 0.05) to recognize significant differences in expression levels. A selection of 141 mapped genes was made from the 613 genes identified, and analysed for expression differences between watered and water withhold plants at various developmental stages. These log fold-change expression data is first subjected to hierarchical clustering and obtained clusters groups was refined by applying a K-means clustering method according to [35]. Heat maps were generated using Genesis software [54]. The differentially expressed genes were functionally assigned according to [35]. Functionally overrepresented gene categories have been calculated by Fisher's exact test with a P-value cut off 0.01 [35].

## List of abbreviations

AB: advanced backcross; DAF: days after fertilization; DW: dry weight; EST: expressed sequence tag; GBS: Gatersleben barley SNP; OWB: Oregon Wolfe Barleys; PCA: principal component analysis; RMA: robust multichip average; SNP: single nucleotide polymorphism; SM: Steptoe × Morex mapping population; TF: transcription factor; TGW: thousand grain weight

## Authors' contributions

SW carried out the molecular genetic analysis, sequence alignment and linkage mapping, and participated in the design of experiments and the drafting of the marker part of the manuscript. CP designed the PCR primers, while RK and VTH performed the glasshouse drought tolerance assessments, measured starch content and isolated RNA. AB, VK and LK provided genetic material and conducted the field-based drought screening. MSR monitored the marker study and co-edited the part of manuscript, along with UW who conceived the study. NS coordinated the work of the GABI-GRAIN consortium, contributed to the development of concepts, conducted gene expression analysis and critically revised the manuscript. All the authors have read and approved the final manuscript.

## Supplementary Material

Additional file 1 **Classification of ESTs according to MapMan functional categories **[35]. Click here for file

Additional file 2 **SNP frequency and the number of mapped markers per population (shown in parenthesis)**. SM: Steptoe-Morex population, OWB: Oregon Wolfe mapping population, Sc_ISR42_8: *H. vulgare *cultivar Scarlett-*H. spontaneum *ISR48 introgression line population, BHS584: *H. vulgare *cultivar Brenda- *H*. spontaneum584 introgression line population. Click here for file

Additional file 3 **Genes whose expression was induced/repressed by drought stress imposed at various stages of development**. Their map location, putative function and normalized expression ratios (control vs drought stressed) are indicated. Statistical significance is indicated as 0: non-significant, -1 significantly up-regulated, +1 significantly down-regulated under drought. Click here for file

Additional file 4 ***De novo *SNP markers**. int. ID: internal identification number, off. ID: official identification number, Chr.: chromosome location, unigene A35: unigene number, according to HarvEST:Barley assembly 35 http://harvest.ucr.edu, EST: identifier taken either from the CR-EST database, or from the Affymetrix Barley Contigs, alt. EST: identifier of alternative (orthologous) ESTs used for primer design, Locusprimer: the sequence of the PCR primers used for re-sequencing, Pyrosequencingprimer: the sequence of the PCR primers used for pyrosequencing. "Redundancy to other SNP maps" indicates that the same gene target is present in one or more of four independent genetic maps; "Genotype" refers to the population tested; "Dispensation order" indicates the dispensation order applied in pyrosequencing. Click here for file

Additional file 5 **Haplotype groups based on observed variation in a set of 17 starch biosynthesis/degradation genes are provided**. Additionally shown are the seed starch content values for each line under drought as well as control conditions. A detailed list of accessions, their origin and IG-number is also supplied. Click here for file

Additional file 6 **Correlations between seed starch content of control and drought stress from the accessions pertaining specific haplotypes in sucrose synthase**. Click here for file

Additional file 7 **Heatmap of Z-score normalized thousand grain weight (TGW) data from drought stress experiments of field-grown (F), rain shelter (RS) from the two consecutive years (2007 and 2008)**. Red colour indicates higher TGW, yellow -medium and blue -lower TGW. Click here for file

Additional file 8 **Detailed list of accessions, their origin and IG-number is provided**. Click here for file
