# Supplementary material for: Haplotyping, linkage mapping and expression analysis of barley genes regulated by terminal drought stress influencing seed quality
Source: BMC Plant Biol. 2011 Jan 4;11:1. doi: 10.1186/1471-2229-11-1 (PMC3025944; doi:10.1186/1471-2229-11-1)
Supplement: Additional file 7 — Heatmap of Z-score normalized thousand grain weight (TGW) data from drought stress experiments of field-grown (F), rain shelter (RS) from the two consecutive years (2007 and 2008). Red colour indicates higher TGW, yellow -medium and blue -lower TGW. [file 1471-2229-11-1-S7.PPT]

## Slide 1
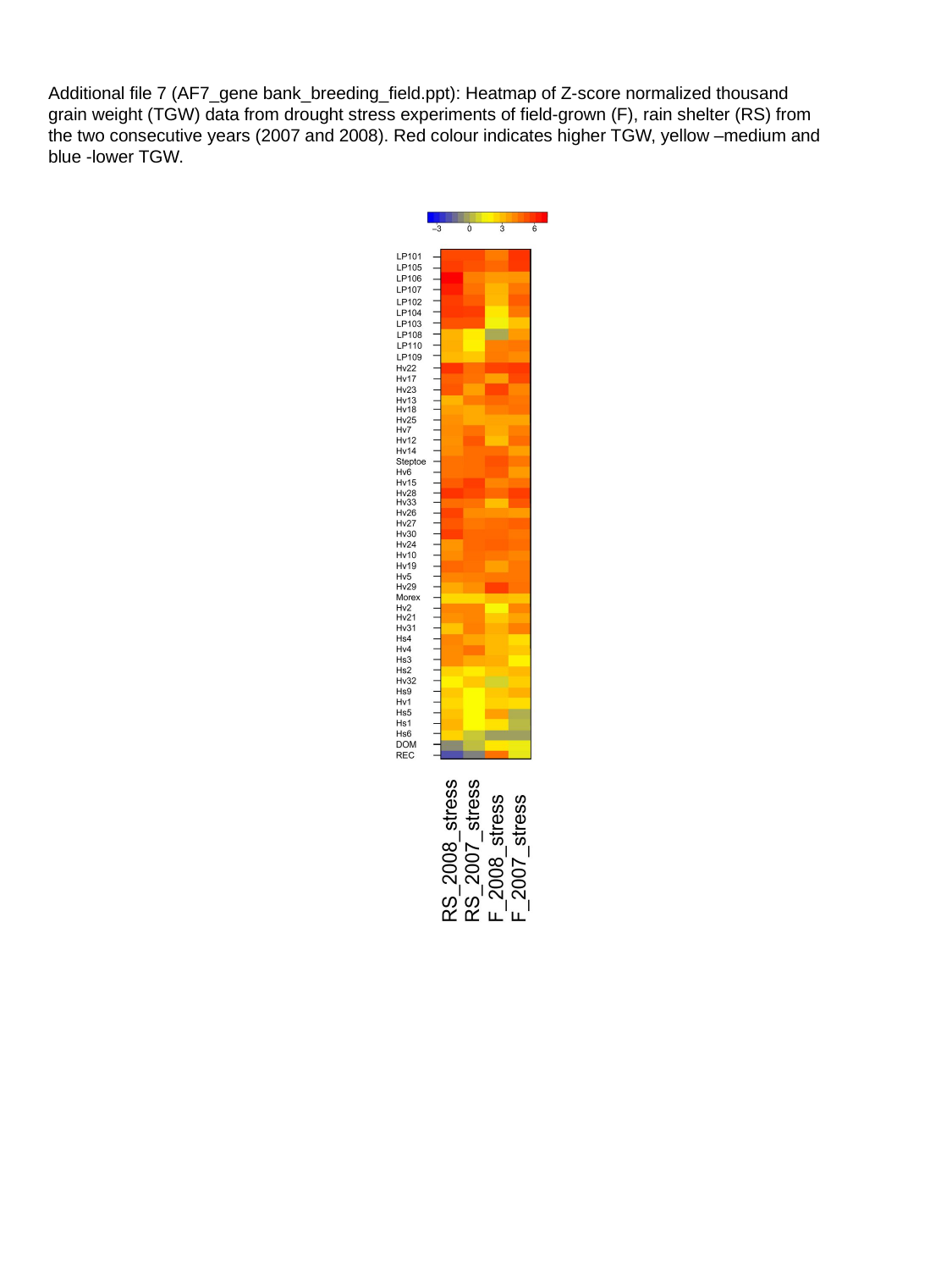

Additional file 7 (AF7_gene bank_breeding_field.ppt): Heatmap of Z-score normalized thousand grain weight (TGW) data from drought stress experiments of field-grown (F), rain shelter (RS) from the two consecutive years (2007 and 2008). Red colour indicates higher TGW, yellow –medium and blue -lower TGW.
