# Supplementary material for: Haplotyping, linkage mapping and expression analysis of barley genes regulated by terminal drought stress influencing seed quality
Source: BMC Plant Biol. 2011 Jan 4;11:1. doi: 10.1186/1471-2229-11-1 (PMC3025944; doi:10.1186/1471-2229-11-1)
Supplement: Additional file 8 — Detailed list of accessions, their origin and IG-number is provided. [file 1471-2229-11-1-S8.PPT]

## Slide 1
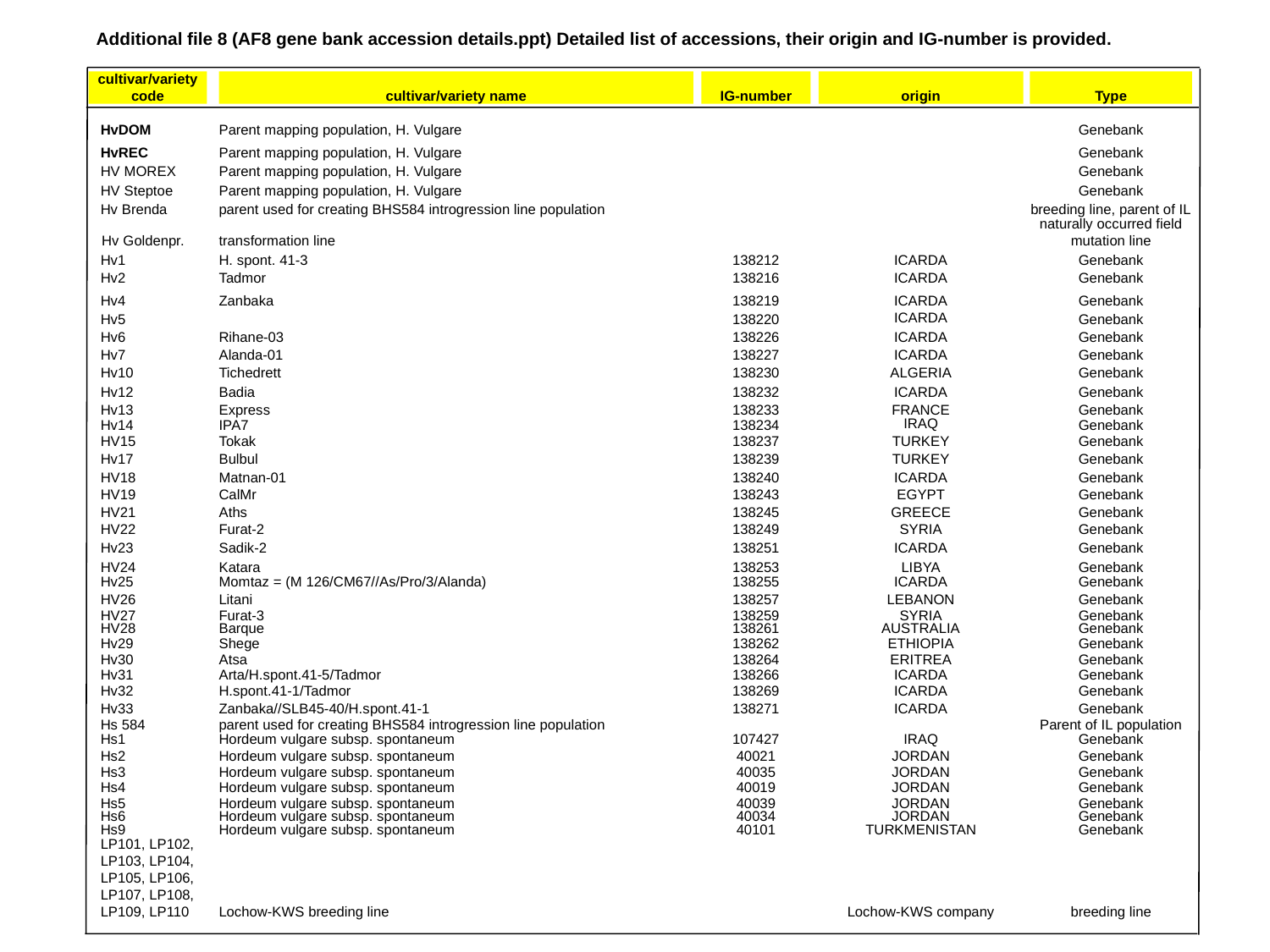

Additional file 8 (AF8 gene bank accession details.ppt) Detailed list of accessions, their origin and IG-number is provided.
cultivar/variety code
cultivar/variety name
IG-number
origin
Type
HvDOM
Parent mapping population, H. Vulgare
Genebank
HvREC
Parent mapping population, H. Vulgare
Genebank
HV MOREX
Parent mapping population, H. Vulgare
Genebank
HV Steptoe
Parent mapping population, H. Vulgare
Genebank
Hv Brenda
parent used for creating BHS584 introgression line population
breeding line, parent of IL
Hv Goldenpr.
transformation line
naturally occurred field mutation line
Hv1
H. spont. 41-3
138212
ICARDA
Genebank
Hv2
Tadmor
138216
ICARDA
Genebank
ICARDA
Hv4
Zanbaka
138219
ICARDA
Genebank
Hv5
138220
Genebank
Hv6
Rihane-03
138226
ICARDA
Genebank
Hv7
Alanda-01
138227
ICARDA
Genebank
Hv10
Tichedrett
138230
ALGERIA
Genebank
Hv12
Badia
138232
ICARDA
Genebank
Hv13
Express
138233
FRANCE
Genebank
IRAQ
Hv14
IPA7
138234
Genebank
HV15
Tokak
138237
TURKEY
Genebank
Hv17
Bulbul
138239
TURKEY
Genebank
HV18
Matnan-01
138240
ICARDA
Genebank
HV19
CalMr
138243
EGYPT
Genebank
HV21
Aths
138245
GREECE
Genebank
HV22
Furat-2
138249
SYRIA
Genebank
Hv23
Sadik-2
138251
ICARDA
Genebank
HV24
Katara
138253
LIBYA
Genebank
Hv25
Momtaz = (M 126/CM67//As/Pro/3/Alanda)
138255
ICARDA
Genebank
HV26
Litani
138257
LEBANON
Genebank
HV27
Furat-3
138259
SYRIA
Genebank
HV28
Barque
138261
AUSTRALIA
Genebank
Hv29
Shege
138262
ETHIOPIA
Genebank
Hv30
Atsa
138264
ERITREA
Genebank
Hv31
Arta/H.spont.41-5/Tadmor
138266
ICARDA
Genebank
Hv32
H.spont.41-1/Tadmor
138269
ICARDA
Genebank
Hs 584
parent used for creating BHS584 introgression line population
Parent of IL population
Hv33
Zanbaka//SLB45-40/H.spont.41-1
138271
ICARDA
Genebank
Hs1
Hordeum vulgare subsp. spontaneum
107427
IRAQ
Genebank
Hs2
Hordeum vulgare subsp. spontaneum
40021
JORDAN
Genebank
Hs3
Hordeum vulgare subsp. spontaneum
40035
JORDAN
Genebank
Hs4
Hordeum vulgare subsp. spontaneum
40019
JORDAN
Genebank
Hs5
Hordeum vulgare subsp. spontaneum
40039
JORDAN
Genebank
Hs6
Hordeum vulgare subsp. spontaneum
40034
JORDAN
Genebank
Hs9
Hordeum vulgare subsp. spontaneum
40101
TURKMENISTAN
Genebank
LP101, LP102, LP103, LP104, LP105, LP106, LP107, LP108, LP109, LP110
Lochow-KWS breeding line
Lochow-KWS company
breeding line
